# Healthcare utilization and costs among prolactinoma patients: a cross-sectional study and analysis of determinants

**DOI:** 10.1007/s11102-020-01089-1

**Published:** 2020-10-06

**Authors:** Merel van der Meulen, Amir H. Zamanipoor Najafabadi, Daniel J. Lobatto, Wilbert B. van den Hout, Cornelie D. Andela, Ingrid M. Zandbergen, Alberto M. Pereira, Wouter R. van Furth, Thea P. M. Vliet Vlieland, Nienke R. Biermasz

**Affiliations:** 1 Department of Medicine, Division of Endocrinology, Pituitary Center and Center for Endocrine Tumors, Leiden University Medical Center, Leiden, The Netherlands; 2 University Neurosurgical Centre Holland, Leiden University Medical Centre, Haaglanden Medical Centre and Haga Teaching Hospital, Leiden, The Hague The Netherlands; 3 Department of Medical Decision Making & Quality of Care, Leiden University Medical Center, Leiden, The Netherlands; 4 Department of Orthopedics, Rehabilitation and Physical Therapy, Leiden University Medical Center, Leiden, The Netherlands

**Keywords:** Pituitary adenoma, Prolactinoma, Healthcare utilization, Costs, Health-related quality of life, Value-based healthcare

## Abstract

**Purpose:**

Prolactinomas are the most prevalent functioning pituitary adenomas. They affect gonadal function as well as health-related quality of life (HRQoL). This study aimed to report healthcare utilization and costs, including their determinants, for prolactinoma patients.

**Methods:**

Cross-sectional study of 116 adult prolactinoma patients in chronic care in a Dutch tertiary referral center. Patients completed four validated questionnaires, assessing healthcare utilization and costs over the previous 12 months (Medical Consumption Questionnaire), disease bother and needs (Leiden Bother and Needs Questionnaire Pituitary), HRQoL (Short Form-36), and self-reported health status (EuroQol 5D). Regression analyses were used to assess associations between disease-related characteristics and healthcare utilization and costs.

**Results:**

Mean age was 52.0 years (SD 13.7) and median follow-up was 15.0 years (IQR 7.6–26.1). Patients visited the endocrinologist (86.2%), general practitioner (37.9%), and ophthalmologist (25.0%) most frequently. Psychological care was used by 12.9% of patients and 5% were admitted to hospital. Mean annual healthcare costs were €1928 (SD 3319), mainly for pituitary-specific medication (37.6% of total costs), hospitalization (19.4%) and specialist care (16.1%). Determinants for higher healthcare utilization and costs were greater disease bother and needs for support, lower HRQoL, elevated prolactin, and longer disease duration, while tumor size, hypopituitarism and adrenal insufficiency were not significantly associated with healthcare utilization and costs.

**Conclusion:**

Healthcare utilization and costs of prolactinoma patients are related to patient-reported HRQoL, bother by disease and needs for support. Therefore, addressing patients’ HRQoL and needs is a way forward to improve efficiency of care and patients’ health status.

**Electronic supplementary material:**

The online version of this article (10.1007/s11102-020-01089-1) contains supplementary material, which is available to authorized users.

## Introduction

Prolactinomas are the most prevalent type of pituitary adenoma, comprising up to 66% of all pituitary adenomas [1]. Beyond the classic hypogonadal symptoms [2], many patients experience cognitive and psychological symptoms [3, 4] that contribute to impairments in health-related quality of life (HRQoL) [5]. Dopamine agonists (DA) are the first-line treatment for most patients [6]. While these are highly effective and generally well tolerated [7], side effects may occur [8], most frequently fatigue (30%), libido changes (28%), and sleep disorders (25%) [9]. Moreover, particularly psychological side effects (e.g., depressive symptoms and behavioral changes) are considered to be underreported [10]. Medical treatment may be temporary, although recent reviews have shown lower remission rates than previously thought (21–36.6%) [9, 11, 12], suggesting the need for long-term medical treatment [11, 12], including ongoing follow-up by endocrinologists and other healthcare professionals [6]. In case of resistance or intolerance to medical therapy, and depending on patient preference, transsphenoidal selective adenomectomy can be considered as alternative treatment option [6, 13].

Considering the long-term treatment and follow-up, substantial healthcare utilization and treatment costs of prolactinoma patients can be anticipated. Three studies [14–16] have used simulation models to compare the cost-effectiveness of DA therapy and surgical treatment for prolactinomas, with cost estimates ranging from $2485 to $6042 per year for medical therapy, and up to $19,224 for the first year after surgery (including admission for surgery) [14–16]. In these studies, the costs of surgically treated patients were highest in the first year after surgery, due to the actual intervention and perioperative care, and decreased over time. Consequently, surgery became more cost-effective than DA after five to ten years of follow-up [14]. However, despite the relatively high prevalence of prolactinomas compared to other pituitary adenomas, no empirical studies have reported the annual healthcare utilization and costs in patients with a prolactinoma, whereas such reports, although scarce, do exist for patients with Cushing’s disease [17–19], acromegaly [20–23], and non-functioning pituitary adenomas (NFA) [24]. Moreover, insight into the determinants of healthcare utilization and costs of prolactinoma patients is lacking, which might provide information relevant to improve efficiency of care and potentially reduce the costs.

Therefore, this study aimed to report the healthcare utilization, healthcare costs, and their determinants for prolactinoma patients. Microprolactinomas treated with DA were a group of particular interest, as microprolactinomas are the most common type of prolactinomas (around 60%) [25] with DA recommended as first-line treatment [6]. We hypothesized that macroprolactinomas were associated with higher healthcare utilization and costs, since these are more often resistant to DA [26, 27], and are more often complicated with visual complaints and hypopituitarism than microprolactinomas [28, 29]. In line with previous findings in patients with an NFA [24], we also expected that patients reporting a greater bother by disease and more needs for support would have higher healthcare utilization and costs. Identification of these disease- or care-related determinants will enhance the understanding of the factors driving healthcare utilization and costs, which can be used to improve efficiency of care and may consequently improve health outcomes of patients treated for a prolactinoma.

## Patients and methods

### Study design

This study presents the data of prolactinoma patients that were collected in a large cross-sectional study assessing healthcare utilization, costs, and work-related disability among pituitary patients [24, 30]. It was performed at the Leiden University Medical Center (LUMC), a tertiary referral center for the diagnosis and treatment of pituitary adenomas in the Netherlands, and was approved by the institutional ethical committee (p12.067).

### Patients

Patients were eligible for participation if they were diagnosed with a prolactinoma (based on symptoms, biochemistry, and MRI), aged ≥ 18 years, had sufficient Dutch language skills, and had visited the outpatient clinic of our center after December 2014. Patients were excluded if they had not received follow-up care in our center after December 2014, were incapable of completing the questionnaires themselves, or were living abroad. Eligible patients were identified from the hospital registry and invited to participate in this study by their treating physician by means of a letter between September 2016 and March 2017. In case of no response, patients were approached a second time by letter. After informed consent was obtained, patients were enrolled in the study.

### Assessments

Patients completed a set of four validated questionnaires either digitally or on paper. Healthcare usage and costs were assessed with the Medical Consumption Questionnaire of the institute for Medical Technology Assessment (iMTA MCQ) [31], perceived bother by disease and needs for support were assessed using the Leiden Bother and Needs Questionnaire for patients with pituitary disease (LBNQ-Pituitary) [32], HRQoL was measured using the Short Form-36 (SF-36) [33, 34], and the EuroQol (EQ-5D) [35] was used to assess utility and self-reported health status.

### Sociodemographic and clinical characteristics

Several clinical and sociodemographic characteristics were collected from the medical records, including age, sex, date of diagnosis, tumor size at diagnosis, treatment, and prolactin level at time of study participation. Tumor size was categorized into (1) microprolactinoma (< 10 mm), (2) macroprolactinoma (10–40 mm), (3) giant prolactinoma (≥ 40 mm), and (4) invisible on MRI. The prolactin level at time of study participation (any prolactin level recorded in the patient’s medical record between August 2016 and May 2017) was classified as elevated (females > 23.0 μg/L, males > 15.0 μg/L) or normal. Since no data on menopausal status were collected, the percentage of female participants in the postmenopausal age range ($\ge$ 50 years) and premenopausal age range (< 50 years) [36] were presented, to give an indication of menopausal status in this cohort.

Self-reported characteristics included marital status, educational level, use of hormone replacement therapy (which was used to determine presence of pituitary dysfunction in any axis), and specifically the use of hydrocortisone replacement (which was used to determine presence of adrenal insufficiency). Level of education was categorized into low, intermediate or high, according to the guidelines of Statistics Netherlands (CBS) [37], that are based on the International Standard Classification of Education: Fields of Training and Education 2013 by UNESCO [38].

### Healthcare utilization

To assess the number of appointments a patient had had with various healthcare professionals in the previous 12 months, the iMTA MCQ [31] was used. We considered appointments with the following healthcare professionals relevant for prolactinoma patients: general practitioner; endocrinologist; neurosurgeon; ophthalmologist; ear, nose and throat (ENT) specialist; neurologist; radiation oncologist; cardiologist; gynecologist; internist; any other medical specialists; occupational physician; psychiatrist/psychologist; physiotherapist; and dietician. Patients were categorized into high (≥ 3 visits) or low specialist care utilization (< 3 visits), based on the median total number of visits to any medical specialist during the previous 12 months. Moreover, the iMTA MCQ was used to assess medication usage (including frequency and dosage), emergency care (i.e. ambulance rides, visits to the emergency department), hospital admissions (including frequency and duration), and home care (i.e. nursing care, household help).

### Perceived bother and needs for support

The LBNQ-Pituitary is a disease-specific questionnaire, that was developed and validated in a large Dutch sample of patients with pituitary disease in the Netherlands [32]. This questionnaire comprises 26 items covering five subscales: mood problems, negative illness perceptions, sexual functioning problems, physical and cognitive complaints, and social functioning problems. For each subscale and for the total questionnaire, an index score can be calculated (range 0–100) for both bother and needs. Higher scores indicate greater bother by the consequences of the disease and greater needs for support [32].

### Health-related quality of life and utility

The SF-36 is a generic HRQoL questionnaire consisting of 36 items covering eight domains: physical functioning, physical role functioning, bodily pain, general health, vitality, social functioning, emotional role functioning and mental health. These subscales can be used to calculate a physical (PCS) and mental component score (MCS). Both the subscales and the component scores range from 0 to 100, with higher scores indicating better HRQoL [33].

The EQ-5D (5-level) consists of 5 domains (mobility, self-care, usual activities, pain/discomfort, and anxiety/depression) with 5 levels (no, slight, moderate, severe, and extreme problems), that are used to calculate utility (range − 0.446 to 1; EQ-5D index). The EQ-5D also includes a visual analogue scale (VAS), on which patients score their perceived health status (range 0–100), with higher scores indicating a better self-reported health status [35].

### Costs

Healthcare costs were calculated based on the healthcare utilization as reported by the patients. Prices were obtained in accordance with the Dutch manual for costing research [39] and were based on reference prices for 2016, which have been published previously [24]. The presented costs can be converted to dollars using the purchasing power parity provided by the Organization for Economic Co-operation and Development (OECD), which was 0.796 per dollar in 2016 [40]. Medical costs, medication costs, and total costs were presented separately. Medical costs include medical costs of chronic specialist care (including the general practitioner, specialist care, occupational care, mental healthcare, and allied health professionals), medical costs of acute care (including ambulance rides, emergency department visits, and hospitalization), and home care costs. Medication costs were presented separately for dopamine agonists (cabergoline, bromocriptine, quinagolide) and hormone replacement therapy (androgel, contraceptives, thyrax, hydrocortisone, genotropin, and desmopressin).

### Statistics

**Fig. 1:**
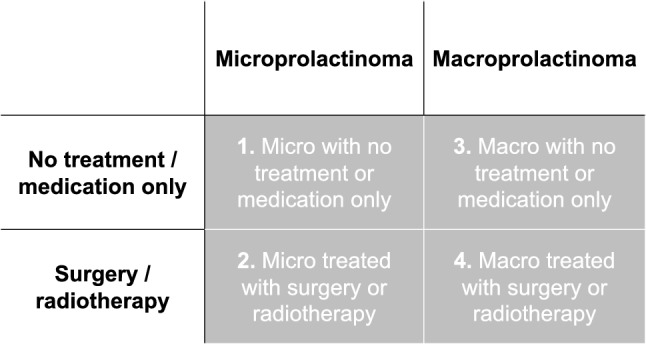
Visualization of the four main subgroups

An online survey platform (NET-Q) was used for the questionnaire data entry and all statistical analyses were performed using IBM SPSS 25.0 software (IBM Corp., Armonk, NY). Baseline characteristics, healthcare utilization, and costs were presented using descriptives for the total prolactinoma cohort and were compared between the following main subgroups: (1) patients with a microprolactinoma treated with DA only or not treated at all, (2) patients with a microprolactinoma treated with surgery and/or radiotherapy, (3) patients with a macroprolactinoma treated with DA only or not treated at all, and (4) patients with a macroprolactinoma treated with surgery and/or radiotherapy (Fig. 1). This categorization was chosen based on clinical grounds to gain insight into the healthcare utilization and costs of the ‘typical’ prolactinoma, the DA-treated microprolactinoma, which may be underrepresented in this academical cohort, since these patients are typically not always referred. These four groups were only compared descriptively for healthcare utilization and costs, as we focused on presenting the absolute costs of these clinically relevant subgroups, rather than statistical differences.

In addition, formal comparisons were made between tumor sizes (micro vs. macro). For the comparisons between tumor sizes, giant prolactinomas were classified as macroprolactinomas, and invisible adenomas were omitted from the analyses (‘missing’). Continuous variables were presented as means and standard deviations (SD) or medians with interquartile ranges (IQR) and were compared within the additional categories using the unpaired t-test or Mann–Whitney U test for normal and skewed distributions, respectively. Categorical variables were presented as frequencies with percentages and comparisons between tumor sizes were made using Chi-square analyses.

Logistic regression analysis was used to determine associations between specialist care utilization (high/low) and possible health-related contributing factors (clinical characteristics, HRQoL, bother by disease, and needs for support), and associations were expressed as odds ratios (ORs) with 95% confidence intervals (CIs) and P-values. Associations between overall healthcare costs and the possible contributing factors were determined using linear regression analysis and were presented as regression coefficients ($\beta$) with corresponding 95% CIs and P-values. In order to control for confounding, a separate multivariable regression analysis was performed for the association between each determinant and the outcome, corrected for confounders. In other words, every multivariable regression analysis involved one determinant and included several potential confounders as covariates. Confounders were defined as variables that were associated with both the determinant and the outcome and were not in the causal pathway [41]. Thus, all associations were corrected for age and sex and depending on the determinant also for treatment type or education level. These regression analyses were performed for the total cohort. In addition, for the variables that were significantly different between microprolactinoma and macroprolactinoma patients, the regression analyses were also stratified for tumor size (micro vs. macro).

Perceived bother by disease and needs for support were analyzed using ANCOVA, correcting for age, sex, and education level (Supplement 4).

For all analyses, a P-value < 0.05 (two-sided) was considered statistically significant. Because of the low number of missing values (< 5%), complete case analysis was used to handle missing questionnaire data.

## Results

### Study population and patient characteristics

**Fig. 2:**
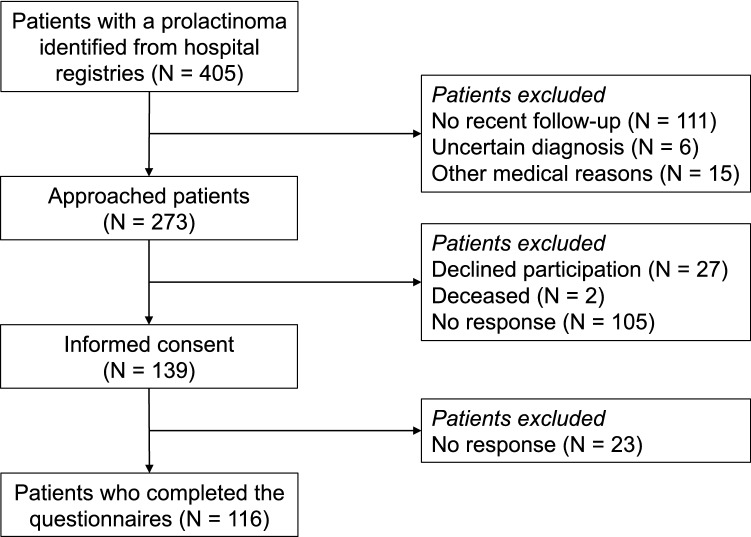
Flowchart of patient inclusion

A total of 405 patients with a possible prolactinoma were identified from the hospital registry. After exclusion of patients without a confirmed diagnosis and those not in follow-up care at our institution, 273 patients were invited for participation, 116 (42%) of whom were enrolled in this study (Fig. 2). The resulting cohort comprised 82 (70.7%) females, 40 of whom (48.8%) were in the postmenopausal age range (50 years or older). The mean age of the cohort was 52.0 years (SD 13.7, median 53.3, IQR 42.4–61.2) and the median time since diagnosis was 15.0 years (IQR = 7.6–26.1) (Table 1). Hypopituitarism was present in 49 patients (42.2%), adrenal insufficiency in 19 patients (16.4%), and 38 patients (32.8%) had persistently elevated prolactin levels at the time of study participation. More than half of the patients (64, 55.2%) were using DA and 28 patients (24.1%) had undergone transsphenoidal surgery. Of the 47 patients with a medically treated microprolactinoma (Table 1), 39 (83.0%) were female, 20 (52.6%) had elevated prolactin levels, 13 (27.7%) had hypopituitarism, and 3 (6.4%) had adrenal insufficiency. The additional comparisons are presented in Supplement 1.

**Table 1:** Characteristics of 116 patients with a prolactinoma, categorized by tumor size and treatment

	Total cohort (N = 116)*	Micro, no treatment or medication only (N = 47)*	Micro, surgery and/or radiotherapy (N = 6)*	Macro, no treatment or medication only (N = 37)*	Macro, surgery and/or radiotherapy (N = 21)*	Missing data of the total cohort, N (%)
Demographic characteristics						
Sex, N (%)						
Female	82 (70.7)	39 (83.0)	6 (100.0)	19 (51.4)	14 (66.7)	0
Age in years, mean (SD)	52.0 (13.7)	51.0 (14.5)	44.8 (16.5)	50.7 (12.7)	56.0 (13.2)	0
Female patients aged $\ge$ 50 years, N (% of female patients)	40 (48.8)	17 (43.6)	2 (33.3)	8 (42.1)	9 (64.3)	0
Marital status, N (%)						1 (0.9)
Relationship/married	87 (75.7)	34 (72.3)	5 (83.3)	29 (80.6)	16 (76.2)	
Education, N (%)						1 (0.9)
Low	30 (26.1)	8 (17.0)	3 (50.0)	12 (33.3)	4 (19.0)	
Intermediate	27 (23.5)	15 (31.9)	1 (16.7)	4 (11.1)	7 (33.3)	
High	58 (50.4)	24 (51.1)	2 (33.3)	20 (55.6)	10 (47.6)	
Disease characteristics						
Time since diagnosis in years, median (IQR)	15.0 (7.6–26.1)	16.6 (9.1–26.5)	17.0 (5.0–27.9)	11.0 (4.0–16.0)	27.0 (10.1–35.5)	0
Tumor size at diagnosis, N (%)						2 (1.7)
Micro (< 10 mm)	53 (46.5)	47 (100.0)	6 (100.0)	0 (94.6)	0 (100.0)	
Macro (10–40 mm)	56 (49.1)	0	0	35 (5.4)	21	
Giant (> 40 mm)	2 (1.8)	0	0	2	0	
Invisible on MRI or CT	3 (2.6)	0	0	0	0	
Treatment, N (%)						0
No treatment	3 (2.6)	3 (6.4)	0	0	0	
Medication only	84 (72.4)	44 (93.6)	0	37 (100.0)	0	
Surgery only	0	0	0	0	0	
Radiotherapy only	0	0	0	0	0	
Medication + surgery	20 (17.2)	0	6 (100.0)	0	14 (66.7)	
Medication + radiotherapy	1 (0.9)	0	0	0	1 (4.8)	
Surgery + radiotherapy	4 (3.4)	0	0	0	3 (14.3)	
Medication + surgery + radiotherapy	4 (3.4)	0	0	0	3 (14.3)	
Endocrine status, N (%)						
Elevated prolactin level	3 (32.8	20 (52.6)	3 (60.0)	8 (21.6)	4 (28.6)	17 (14.7)
Hypopituitarism	4 (42.2)	13 (27.7)	1 (16.7)	18 (48.6)	14 (66.7)	
Adrenal insufficiency	1 (16.4)	3 (6.4)	0	7 (18.9)	7 (33.3)	
Self-reported health status						
EQ-5D score, mean (SD)^	0.91 (0.083)	0.908 (0.099)	0.906 (0.114)	0.925 (0.060)	0.901 (0.079)	0
EQ-5D VAS, mean (SD)^	75.6 (20.3)	73.6 (21.8)	71.7 (21.1)	78.7 (18.4)	76.7 (16.1)	0
SF-36 PCS, mean (SD)^	49.0 (9.8)	48.5 (10.6)	48.4 (10.0)	51.3 (8.1)	45.3 (10.7)	0
SF-36 MCS, mean (SD)^	48.1 (11.7)	47.8 (12.2)	46.5 (11.6)	49.0 (11.4)	48.0 (12.0)	0
LBNQ-Pituitary Bother by disease, total index score, mean (SD)†	15.8 (19.1)	14.5 (18.5)	26.9 (29.2)	13.4 (17.3)	19.9 (20.5)	5 (4.3)
LBNQ-Pituitary needs for support, total index score, mean (SD)†	16.7 (20.3)	16. (20.4)	29.6 (33.3)	13.3 (17.9)	20.1 (19.9)	8 (6.9)

Due to rounding, not all percentages of the categorical variables add up to 100%

*N* number, *SD* standard deviation, *IQR* interquartile range, *RT* radiotherapy, *VAS* visual analogue scale, *EQ-5D* EuroQoL, *SF-36* Short Form-36, *LBNQ-Pituitary* Leiden Bother and Needs Questionnaire for patients with pituitary disease, *MCS* mental component score, *PCS* physical component score

*Percentages are calculated over the number of patients for whom data were available per variable

^Higher scores indicate better HRQoL

^†^Lower scores indicate lower disease burden

### Healthcare utilization

#### Primary care

The general practitioner had been consulted by 44 patients (37.9%) in the previous year, and by 19 (40.4%) of the patients with medically treated microprolactinomas (Table 2). Of the other primary healthcare professionals, the physiotherapist had been visited most frequently (total cohort: n = 22, 19.0%; medically treated microprolactinoma: n = 10, 21.3%). Additional comparisons can be found in Supplement 2.

**Table 2:** Average healthcare utilization over the past 12 months among 116 patients with a prolactinoma, categorized by tumor size and treatment

Healthcare service	Total cohort (N = 116)		Micro, no treatment or medication only (N = 47)		Micro, surgery and/or radiotherapy (N = 6)		Macro, no treatment or medication only (N = 37)		Macro, surgery and/or radiotherapy (N = 21)	
	Number of patients (%)	Visits among those visiting, mean (SD)	Number of patients, %	Visits among those visiting, mean (SD)	Number of patients, %	Visits among those visiting, mean (SD)	Number of patients, %	Visits among those visiting, mean (SD)	Number of patients, %	Visits among those visiting, mean (SD)
General practitioner	44 (37.9)	4.3 (4.9)	19 (40.4)	4.1 (4.5)	2 (33.3)	2.5 (0.7)	10 (27.0)	5.1 (5.2)	10 (47.6)	4.9 (6.4)
Pituitary adenoma-related medical specialists										
Endocrinologist	100 (86.2)	2.0 (1.5)	40 (85.1)	2.0 (1.6)	3 (50.0)	3.7 (3.8)	34 (91.9)	2.1 (1.4)	19 (90.5)	1.6 (0.9)
Neurosurgeon	6 (5.2)	1.3 (0.5)	1 (2.1)	1.0†	1 (16.7)	2.0†	2 (5.4)	1.5 (0.7)	2 (9.5)	1.0 (0.0)
Ophthalmologist	29 (25.0)	2.2 (2.6)	11 (23.4)	2.64 (4.2)	3 (50.0)	2.0 (1.0)	11 (29.7)	2.1 (0.7)	4 (19.0)	1.5 (0.6)
ENT specialist	4 (3.4)	1.8 (1.0)	1 (2.1)	1.0†	0†	–	2 (5.4)	1.5 (0.7)	1 (4.8)	3.0†
Neurologist	7 (6.0)	1.4 (0.8)	4 (8.5)	1.5 (1.0)	1 (16.7)	1.0†	2 (5.4)	1.5 (0.7)	0	–
Radiation oncologist	17 (14.7)	1.3 (0.6)	5 (10.6)	1.6 (0.9)	2 (33.3)	1.5 (0.7)	5 (13.5)	1.2 (0.5)	5 (23.8)	1.0 (0.0)
Cardiologist	13 (11.2)	1.9 (1.6)	4 (8.5)	3.0 (2.7)	1 (16.7)	1.0	5 (13.5)	1.4 (0.6)	3 (14.3)	1.3 (0.6)
Gynecologist	1 (0.9)	1†	1 (2.1)	1.0†	0	–	0	–	0	–
Internist	8 (6.9)	3.5 (2.2)	6 (12.8)	3.5 (2.1)	0	–	1 (2.7)	6.0†	1 (4.8)	1.0†
Others	8 (6.9)	3.1 (1.6)	4 (8.5)	2.5 (1.3)	0	–	2 (5.4)	4.5 (0.7)	2 (9.5)	3.0 (2.8)
Total number of different specialists										
0	10 (8.6)	–	4 (8.5)	–	2 (33.3)	–	1 (2.7)	–	2 (9.5)	–
1	57 (49.1)	1.6 (1.2)	26 (55.3)	1.7 (1.4)	0	–	20 (54.1)	1.6 (1.1)	7 (33.3)	1.3 (0.5)
2	25 (21.6)	4.0 (3.3)	8 (17.0)	5.3 (5.2)	3 (50.0)	3.0 (1.7)	6 (16.2)	4.8 (1.7)	8 (38.1)	2.5 (0.8)
3	12 (10.3)	6.3 (3.1)	5 (10.6)	6.2 (2.7)	0	–	6 (16.2)	6.3 (3.8)	1 (4.8)	7.0†
4 or more	12 (10.3)	10.2 (4.4)	4 (8.5)	13.0 (5.4)	1 (16.7)	15.0†	4 (10.8)	8.5 (2.4)	3 (14.3)	7.0 (2.6)
Occupational care										
Occupational physician	9 (7.8)	5.8 (3.6)	5 (10.6)	6.6 (4.2)	1 (16.7)	5.0†	2 (5.6)	4.0 (2.8)	1 (4.8)	NA
Mental healthcare										
Psychologist/psychiatrist	15 (12.9)	10.3 (7.2)	9 (19.1)	11.8 (7.9)	0	–	3 (8.1)	6.0 (2.0)	3 (14.3)	10.0 (8.7)
Allied health professionals										
Physiotherapist	22 (19.0)	14.3 (18.4)	10 (21.3)	21.3 (24.9)	2 (33.3)	2.5 (2.1)	2 (5.4)	4.5 (0.7)	8 (38.1)	10.9 (7.8)
Dietician	8 (6.9)	4.0 (3.3)	5 (10.6)	3.2 (3.8)	0	–	2 (5.4)	6.0 (2.8)	1 (4.8)	4.0†
Total number of different allied health professionals										
0	89 (76.7)	–	34 (72.3)	–	4 (66.7)	–	33 (89.2)	–	13 (61.9)	–
1	24 (20.7)	8.6 (6.5)	11 (23.4)	10.6 (7.6)	2 (33.3)	2.5 (2.1)	4 (10.8)	5.3 (1.9)	7 (33.3)	9.0 (6.1)
2	3 (2.6)	46.7 (39.5)	2 (4.3)	56.0 (50.9)	0	–	0	–	1 (4.8)	28.0†
Emergency care										
Ambulance rides	5 (4.3)	1.0 (0.0)	3 (6.4)	1.0 (0.0)	1 (16.7)	1.0†	1 (2.7)	1.0†	0	–
Emergency department visit(s)	12 (10.3)	1.3 (0.5)	6 (12.8)	1.2 (0.4)	2 (33.3)	1.5 (0.7)	2 (5.4)	1.5 (0.7)	2 (9.5)	1.0 (0.0)
Hospital admission(s)	6 (5.1)	8.8 (5.7)*	2 (4.3)	6.5 (0.7)*	2 (33.3)	8.0 (0.0)*	1 (2.7)	20.0†*	1 (4.8)	4.0†*
Home care										
Community nurse	1 (0.9)	13.0†^	1 (2.1)	13.0†^^^	0	–	0	–	0	–
Informal care	0	–	0	–	0	–	0	–	0	–
Household help	2 (1.7)	156.0 (73.5)^	2 (4.3)	156.0 (73.5)^^^	0	–	0	–	0	–

*N* number, *SD* standard deviation, *NA* not available, *ENT* ear, nose and throat

^†^No SD could be calculated because the number of patients in the categories was too low

*Hospital admissions are presented in days

^Community nurse, informal care, and household help are presented in hours

#### Specialist care

The majority of patients (n = 106, 91.4%) had consulted a medical specialist in the previous year, most commonly the endocrinologist (n = 100, 86.2%), the ophthalmologist (n = 29, 25.0%), and the radiation oncologist (n = 17, 14.7%) (Table 2). Medically treated microprolactinoma patients had visited the endocrinologist (n = 40, 85.1%), ophthalmologist (n = 11, 23.4%), and internist (n = 6, 12.8%) most often.

#### Mental healthcare

Psychological/psychiatric care physicians had been visited by 15 patients (12.9%) of the total cohort, and by 9 (19.1%) of the medically treated microprolactinoma patients, which was the highest percentage of the main subgroups (range: 0–19.1%).

#### Hospital admissions and emergency care

In the previous year, 12 patients (10.3%) had visited the emergency department at least once (mean 1.3), and 5 patients (4.3%) had had an ambulance ride (mean 1) (Table 2). Furthermore, 6 patients (5.1%) had been admitted to the hospital at least once, with a mean duration of 8.8 days (range 4–20 days). Of the medically treated microprolactinoma patients, 6 (12.8%) had visited the emergency department, 3 (6.4%) had had an ambulance ride, and 2 (4.3%) had been admitted to the hospital at least once.

#### Determinants of healthcare utilization

After correction for relevant confounders, patients with a longer time since diagnosis (OR 0.954, 95% CI 0.882; 0.976), and patients with a better physical (OR 0.952, 95% CI 0.915; 0.992) and mental (OR 0.953, 95% CI 0.920; 0.986) HRQoL according to the SF-36 had lower specialist care utilization. Conversely, patients with elevated prolactin levels (OR 2.661, 95% CI 1.112; 6.366), greater bother by disease (OR 1.053, 95% CI 1.024; 1.082) or greater needs for support (LBNQ-Pituitary) (OR 1.057, 95% CI 1.028; 1.087) had a higher specialist care utilization (Table 4). When stratified for tumor size, elevated prolactin levels were a significant determinant of healthcare utilization only in microprolactinoma patients (OR 6.318, 95% CI 1.429; 27.934). Overall, tumor size, prolactinoma treatment, hypopituitarism, and adrenal insufficiency were not significantly associated with healthcare utilization.

### Costs

The mean annual healthcare costs of prolactinoma patients who had used healthcare in the previous year were €1928 (SD = 3319) (Table 3). Pituitary-specific medication constituted the largest expenditure (37.6% of all costs), followed by hospitalization (19.4%) and specialist care (16.1%) (Fig. 3). Medication costs were especially high for surgically treated macroprolactinoma patients (€2506), compared to surgically treated microprolactinoma patients (€20), and medically treated microprolactinoma (€552) and macroprolactinoma patients (€764). The main contributor to these high medication costs were the costs for hormone replacement therapy, which were significantly higher for patients with a macroprolactinoma (€1595) compared to patients with a microprolactinoma (€174, P < 0.001) (Supplement 3). Furthermore, medical costs of chronic specialist care and acute care (excluding home care, since this large expenditure was only used by two patients and was probably not prolactinoma-related) were almost 50% higher for patients with microprolactinomas (€1242) compared to macroprolactinomas (€882), although this was not statistically significant (P = 0.441) (Supplement 3).

**Table 3:** Direct and indirect costs (mean and SD) in euros (€) over the past 12 months in 116 patients with a prolactinoma, categorized by tumor size and treatment (missing: n = 5)

Medical costs	Total cohort (N = 116)		Micro, no treatment or medication only (N = 47)		Micro, surgery and/or radiotherapy (N = 6)		Macro, no treatment or medication only (N = 37)		Macro, surgery and/or radiotherapy (N = 21)	
	Number of patients (%)	Costs among those visiting, mean (SD)	Number of patients (%)	Costs among those visiting, mean (SD)	Number of patients (%)	Costs among those visiting, mean (SD)	Number of patients (%)	Costs among those visiting, mean (SD)	Number of patients (%)	Costs among those visiting, mean (SD)
Medical costs of chronic specialist care										
General practitioner	43 (37.1)	141 (138)	19 (40.4)	138 (147)	2 (33.3)	83 (23)	10 (27.0)	168 (171)	10 (47.6)	162 (212)
Specialist care	106 (91.4)	332 (343)	43 (91.5)	358 (411)	4 (66.7)	546 (561)	36 (97.3)	334 (292)	19 (90.5)	273 (221)
Occupational care	9 (7.8)	190 (118)	5 (10.6)	218 (139)	1 (16.7)	165†	2 (5.6)	132 (93)	1 (4.8)	NA
Mental healthcare^	15 (12.9)	657 (464)	9 (19.1)	758 (507)	0	–	3 (8.1)	384 (128)	3 (14.3)	640 (554)
Allied health professionals*	27 (23.3)	423 (577)	13 (27.7)	581 (777)	2 (33.3)	83 (70)	4 (10.8)	173 (62)	8 (38.1)	375 (290)
Total costs of chronic specialist care	111 (95.7)	577 (725)	44 (93.6)	758 (966)	5 (83.3)	536 (641)	36 (97.3)	439 (430)	21 (100.0)	558 (560)
Medical costs of acute care										
Ambulance rides	5 (4.3)	515 (0)	3 (6.4)	515 (0)	1 (16.7)	515†	1 (2.7)	515†	0	–
Emergency department visits	12 (10.3)	324 (117)	6 (12.8)	302 (106)	2 (33.3)	389 (183)	2 (5.4)	389 (183)	2 (9.5)	259 (0)
Hospitalization	6 (5.2)	7061 (6361)	2 (4.3)	3094 (337)	2 (33.3)	7616 (0)	1 (2.7)	19,040†	1 (4.8)	1904†
Total costs of acute care	14 (12.1)	3487 (5373)	6 (12.8)	1591 (1593)	3 (50.0)	5508 (3878)	2 (5.4)	10,166 (13,644)	3 (14.3)	807 (950)
Home care costs										
Home care^#^	2 (1.7)	11,518 (5552)	2 (4.3)	11,518 (5552)	0	–	0	–	0	–
*Medical costs of chronic specialist and acute care*	112 (96.6)	1008 (2352)	45 (95.7)	953 (1418)	5 (83.3)	3841 (4469)	36 (97.3)	1004 (3342)	21 (100.0)	674 (626)
*Total medical costs*	112 (96.6)	1214 (3197)	45 (95.7)	1465 (3707)	5 (83.3)	3841 (4469)	36 (97.3)	1004 (3342)	21 (100.0)	674 (626)

Medication costs	Number of patients (%)	Costs among those using medication, mean (SD)	Number of patients (%)	Costs among those using medication, mean (SD)	Number of patients (%)	Costs among those using medication, mean (SD)	Number of patients (%)	Costs among those using medication, mean (SD)	Number of patients (%)	Costs among those using medication, mean (SD)
Cabergoline	49 (42.2)	526 (616)	17 (36.2)	700 (888)	0	–	26 (70.3)	435 (373)	6 (28.6)	436 (513)
Quinagolide	12 (10.3)	318 (144)	8 (17.0)	323 (110)	0	–	2 (5.4)	322 (379)	2 (9.5)	298 (0)
Bromocriptine	4 (3.4)	62 (61)	3 (6.4)	77 (65)	0	–	0	–	1 (4.8)	19†
*Total costs of dopamine agonists*	64 (55.2)	467 (555)	27 (57.4)	545 (733)	0	–	28 (75.7)	427 (367)	9 (42.9)	359 (430)
Androgel	14 (12.1)	383 (221)	3 (6.4)	428 (383)	0	–	8 (21.6)	392 (173)	3 (14.3)	311 (232)
Contraceptives	5 (4.3)	38 (29)	2 (4.3)	25 (5)	0	–	2 (5.4)	25 (4)	1 (4.8)	90†
Thyrax	31 (26.7)	34 (10)	6 (12.8)	33 (8)	0	–	11 (29.7)	37 (12)	11 (52.4)	31 (10)
Hydrocortisone	19 (16.4)	418 (346)	3 (6.4)	294 (234)	0	–	7 (18.9)	395 (446)	7 (33.3)	385 (84)
Genotropin	11 (9.5)	3333 (1521)	0	–	0	–	2 (5.4)	2298 (243)	9 (42.9)	3564 (1599)
Desmopressin	3 (2.6)	262 (410)	0	–	1 (16.7)	20†	1 (2.7)	31†	1 (4.8)	736†
*Total costs of hormone replacement therapy*	47 (40.5)	1106 (1679)	13 (27.7)	186 (292)	1 (16.7)	20†	17 (45.9)	646 (748)	13 (61.9)	2836 (2296)
*Total medication costs*	81 (69.8)	1011 (1381)	31 (66.0)	552 (703)	1 (16.7)	20†	30 (81.1)	764 (713)	16 (76.2)	2506 (2257)
*Overall costs*	113 (97.4)	1928 (3319)	45 (95.7)	1846 (3676)	5 (83.3)	3845 (4475)	37 (100.0)	1596 (3393)	21 (100.0)	2583 (2243)

*N* number, *SD* standard deviation, *NA* not available

^Psychiatrists and psychologists

*Physiotherapists and dieticians

#Community nurse, informal care, and household help

**Fig. 3:**
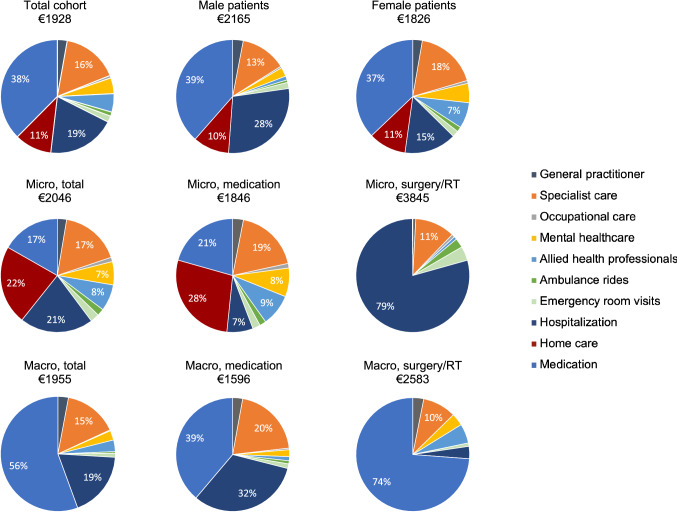
Pie charts showing the proportions of total medical costs for the different expenditures, categorized by tumor size and treatment, and by sex. Micro (microprolactinoma), macro (macroprolactinoma), RT (radiotherapy). Allied health professionals include physiotherapists and dieticians. Percentages are shown for categories contributing more than 5% to the total medical costs

#### Determinants of increased costs

Healthcare costs were not significantly associated with clinical determinants (Table 4). Patients with a better overall physical HRQoL (SF-36) (β =  − 159, 95% CI − 216; − 102) and a higher self-reported health status (EQ VAS) (β =  − 50, 95% CI − 79; − 20) had significantly lower healthcare costs. In contrast, healthcare costs were significantly higher for patients with greater bother by disease (β = 64, 95% CI 31; 97) and greater needs for support (LBNQ-Pituitary) (β = 59, 95% CI 28; 91). In the analysis stratified for tumor size, hypopituitarism was associated with higher costs only in macroprolactinoma patients (β = 1914, 95% CI 197; 3631).

**Table 4:** Logistic/linear regression analysis per determinant for medical specialist utilization and costs among patients with a prolactinoma (N = 116)

Determinant	High specialist utilization (adjusted for demographics*)			Healthcare costs (adjusted for demographics*)		
	OR	95% CI	P-value	$\upbeta$	95% CI	P-value
Clinical determinants						
Time since diagnosis^1,2^	**0.954**	**0.913; 0.996**	**0.033**	21	− 45; 86	0.536
Tumor size at diagnosis (reference: Micro)^1,2^						
Macro (including Giant)	1.033	0.456; 2.298	0.936	− 93	− 1431; 1245	0.891
Treatment (reference: No treatment)^1,2^						
Medication only	1.318	0.109; 16.007	0.828	164	− 3838; 4165	0.936
Medication + surgery	0.774	0.055; 10.800	0.849	1202	− 2998; 5403	0.572
Medication + radiotherapy	0.000	0.000; –	1.000	2614	− 5075; 10,302	0.502
Surgery + radiotherapy	1.996	0.090; 44.289	0.662	530	− 4548; 5607	0.836
Medication + surgery + radiotherapy	0.530	0.019; 14.952	0.709	905	− 4221; 6031	0.727
Endocrine status (reference: Normal/no deficits)^1,2,3^						
Elevated prolactin level	**2.661**	**1.112; 6.366**	**0.028**	− 720	− 1870; 431	0.217
Hypopituitarism	1.086	0.488; 2.418	0.840	1093	− 261; 2447	0.112
Adrenal insufficiency	1.217	0.405; 3.657	0.726	754	− 1135; 2642	0.431
HRQoL, bother by disease and needs for support						
SF-36^1,2,4^						
Mental component scale	**0.954**	**0.921; 0.989**	**0.010**	− 43	− 97; 11	0.115
Physical component scale	**0.949**	**0.911; 0.989**	**0.012**	** − 159**	** − 216; − 102**	** < 0.001**
EQ-5D^1,2,4^						
EQ VAS (scale 0–100)	0.991	0.972; 1.009	0.319	** − 50**	** − 79; − 20**	**0.001**
Bother by disease (LBNQ-Pituitary)^1,2,4^						
Physical & cognitive complaints	**1.040**	**1.019; 1.062**	** < 0.001**	**59**	**33; 84**	** < 0.001**
Mood	**1.042**	**1.019; 1.064**	** < 0.001**	**44**	**18; 69**	**0.005**
Negative illness perceptions	**1.053**	**1.023; 1.085**	**0.001**	31	− 5; 68	0.086
Sexual functioning	**1.020**	**1.002; 1.039**	**0.032**	**52**	**26; 79**	** < 0.001**
Social functioning	**1.038**	**1.013; 1.064**	**0.003**	**36**	**8; 65**	**0.013**
Total index score	**1.054**	**1.025; 1.085**	** < 0.001**	**64**	**31; 97**	** < 0.001**
Needs for support (LBNQ-Pituitary)^1,2,4^						
Physical & cognitive complaints	**1.042**	**1.021; 1.064**	** < 0.001**	**54**	**30; 78**	** < 0.001**
Mood	**1.036**	**1.017; 1.055**	** < 0.001**	**35**	**12; 59**	**0.003**
Negative illness perceptions	**1.047**	**1.021; 1.073**	** < 0.001**	21	− 8; 50	0.152
Sexual functioning	**1.017**	**1.001; 1.034**	**0.039**	**50**	**27; 72**	** < 0.001**
Social functioning	**1.042**	**1.015; 1.069**	**0.002**	**40**	**11; 68**	**0.007**
Total index score	**1.058**	**1.028; 1.088**	** < 0.001**	**59**	**28; 91**	** < 0.001**

**Bold** (P < 0.05), *OR* odds ratio, *CI* confidence interval, *HRQoL* Health related-quality of life, *SF-36* Short Form-36, *EQ-5D* EuroQoL, *LBNQ-Pituitary* Leiden Bother and Needs Questionnaire for patients with pituitary disease

SF-36, EQ-5D: higher scores indicate better HRQoL. LBNQ-Pituitary: lower scores indicate lower bother by disease or needs for support

*^1,2,3,4^Adjusted for age (1), sex (2), treatment (3), education level (4)

## Discussion

Although an increasing number of studies has shown persistent impairments in HRQoL in patients with prolactinomas and other pituitary adenomas [5], little is known about the long-term pituitary disease burden in terms of healthcare utilization and costs. The present study provides insight into the healthcare utilization and costs of prolactinoma patients and shows that the endocrinologist is visited most frequently, reflecting the chronic endocrine care needed for this condition, while other specialists are consulted less frequently. Pituitary-specific medication constitutes the main expenditure. Contrary to our hypothesis, prolactinoma-related healthcare utilization and costs were overall not significantly higher for patients with a macroprolactinoma, except for medication costs. As expected, worse HRQoL, greater bother by disease and greater needs for support were significantly associated with higher healthcare utilization and costs. Besides elevated prolactin levels, no other clinical factors showed significant associations. This accentuates the relevance of attention for HRQoL and patient-reported bother and needs in pituitary care, which can be facilitated by patient-reported outcome measures (PROMs) such as the LBNQ-Pituitary [32]. Furthermore, these findings suggest that interventions aiming to improve HRQoL, such as patient education, psychosocial support, and rehabilitation programs [42–46], can be helpful in reducing healthcare utilization and potentially costs in other areas of healthcare.

No previous empirical studies were available for comparison of healthcare utilization and costs for patients with a prolactinoma. However, three cost simulation studies [14–16] estimated higher annual costs ($2485 to $6042 for medical therapy, and up to $19,224 for the first year following surgery) compared to the costs observed in our population. Compared to other types of pituitary adenomas, healthcare costs of prolactinomas appear to be lower, as previous studies reported costs ranging between $26,269 and $34,992 for Cushing’s disease, between €9200 and €32,807 for acromegaly, and between €3040 and $13,708 for patients with an NFA [17–24]. It is important to note that international comparisons between healthcare costs are hampered by variations in healthcare costs between countries, with especially high healthcare costs in the USA [47], although the few studies performed in European countries showed higher healthcare costs [21, 23, 24] than our study, too. Besides differences in pharmaceutical costs, healthcare expenditures may also be affected by differences between healthcare systems, specifically with regards to the role of general practitioners who serve as gatekeepers for specialist care in the Netherlands [48, 49]. Compared to the general Dutch population, with average yearly healthcare costs of €5129 per capita in 2017 [50], the costs for the prolactinoma patients in our study appear to be lower as well. However, the value of this comparison is limited since we mainly assessed prolactinoma-related costs in our study, while it is likely that patients also had healthcare costs besides these prolactinoma-related costs.

Moreover, we found a lower healthcare utilization in prolactinoma patients than previously reported in other types of pituitary adenoma, as shown by a lower frequency of visits to medical specialists (91.4% vs. 94–99%), hospitalizations (5.1% vs. 9.5–38.4%), and ER visits (10.3% vs. 11.4–34.2%) [17–24]. Compared to the general Dutch population, visits to general practitioners and physiotherapists and hospital admissions reported by prolactinoma patients are also lower [51], but this comparison is subject to the same limitations as described above. Interestingly, however, we found a higher frequency of prolactinoma-related mental healthcare utilization than reported for the general population (9.1%), which was especially remarkable for the ‘typical’ microprolactinoma (19.1%). Furthermore, prolactinoma patients visited medical specialists more frequently (91.4%) than the general population (39.4%) [51], which is in line with expectations because standard care for most prolactinoma patients in our center involves a one- to three-yearly visit to the endocrinologist, and other medical specialists if needed.

The results of the present study were most comparable to our recent healthcare utilization study in patients with an NFA from the same Dutch tertiary referral center, in which we reported total annual healthcare costs of €3040, 13.8% hospitalizations, 6% ambulance rides, and 11.4% ER visits [24]. In accordance with our findings in prolactinoma patients, patients with an NFA visited the endocrinologist (94.6%), ophthalmologist (58.4%), and general practitioner (51.5%) most often [24]. While most other studies did not specify the frequency of visits per healthcare provider, one study in patients with acromegaly [20] reported that primary care and endocrine specialist care were used most frequently.

This is one of the few studies [20, 24] assessing the determinants of healthcare utilization and costs of pituitary adenomas. In contrast to a study in patients with acromegaly that reported that female sex, younger age, hypopituitarism, and a higher number of comorbidities were associated with higher healthcare costs and utilization [20], we only identified elevated prolactin levels as clinical determinant of healthcare utilization, and we did not identify any clinical determinants of healthcare costs. In this cohort, prolactin levels had not completely normalized in almost a third of the patients. Although marginally elevated prolactin levels do not necessary affect patients negatively and may even be beneficial for metabolism [52], we show that elevated prolactin levels are associated with higher healthcare utilization in patients with a prolactinoma. The exact reasons for the elevated prolactin levels in this cohort are unknown, although it is likely that a combination of multiple factors has played a role, including non-adherence, side effects, resistance, and a lack of clinical indication for dopamine agonist treatment. Mainly patients who have started medical therapy recently and patients with resistance to medication have considerably elevated prolactin levels and require more intensive monitoring, which may explain the higher healthcare utilization for patients with elevated prolactin levels.

It may be surprising that no other clinical variables, such as hypopituitarism and adrenal insufficiency, were significantly associated with healthcare utilization and costs. Subgroup analysis revealed that even in macroprolactinoma patients, who suffered most from these complications (Supplement 1), no consistent associations were present. However, we did observe that medication costs were higher for patients with a macroprolactinoma, while medical costs were lower, resulting in similar costs for micro- and macroprolactinomas.

Our finding that lower self-reported HRQoL and greater bother by disease and needs for support were associated with higher healthcare utilization and costs is in line with our previous study in patients with an NFA [24]. Although much remains to be elucidated, it is likely that impairment in HRQoL in patients treated for a prolactinoma is caused by an interplay of symptoms of hyperprolactinemia, side effects of medical therapy, and possibly consequences of hypopituitarism [5]. Moreover, psychosocial interventions such as self-management and educational programs have proven beneficial for pituitary patients’ HRQoL [42–44]. Although these interventions will require financial investment, the relation between HRQoL and healthcare utilization and costs found in NFA and prolactinoma patients suggests that these interventions may not only improve HRQoL but may thereby also reduce costs in other areas of healthcare on the long term. Therefore, more research is needed to identify treatable factors that contribute to the reduced HRQoL in this population, and to develop specific interventions aiming to improve HRQoL.

### Strengths and limitations

This is the first study to report healthcare utilization and costs and their determinants for prolactinoma patients. A clear strength of this study is the fact that self-reported data were used, which is considered the most preferable method for the measurement of healthcare costs [53]. However, the self-reported data also pose a limitation to this study, as patients may not be familiar with medical terminology, such as names of healthcare professionals, and may make mistakes, for example when reporting dosages of medication. Furthermore, while the questionnaire explicitly asked for only prolactinoma-related visits to healthcare professionals and home care, some patients may have overlooked that, since two patients reported high home care utilization which is unlikely to be directly related to their prolactinoma. We were also unable to distinguish between prolactinoma-related and -unrelated hospital admissions and emergency care, as this distinction was not made by the questionnaire that was used. Another limitation is the fact that only pituitary-specific medication was recorded in this study, which may have resulted in an underestimation of total medication costs. Moreover, despite the fact that comorbidities can have important impact on HRQoL [54], no comorbidities were documented in this study and we were therefore unable to analyze comorbidity-specific impact on patients’ HRQoL. Furthermore, while it would have provided valuable information, we decided not to consider giant prolactinomas and invasive prolactinomas separately, since the numbers of patients in these groups were too small to provide representative results. Also, the cross-sectional study design carries a risk of reverse causality in the interpretation of the data, and, as mentioned previously, the international variation in healthcare costs limits the international comparability of our results. Finally, the generalizability of our results may be limited by the single-center design and the fact that only patients who had visited the outpatient endocrine clinic of our tertiary referral center in the past two years were invited for participation. The resulting academic cohort comprises a relatively high percentage of macroprolactinomas, which are more often resistant to medication [26, 27] and were more often surgically treated (Supplement 1) than microprolactinomas. The fact that only 42% of the invited participants provided informed consent may have strengthened this selection bias, and we may have missed some prolactinoma patients who were treated primarily by the gynecologist and had not been referred to the endocrinologist. Although we stratified the analyses for tumor size and treatment in order to gain insight into the common DA-treated microprolactinoma patients, these patients are still a selected cohort treated in a tertiary referral center and are consequently not entirely representative for the general prolactinoma population. New studies in less selected, more representative cohorts of prolactinoma patients are therefore needed to gain insight into the costs of the different treatment strategies for prolactinomas.

## Conclusion

Healthcare utilization and costs of patients treated for a prolactinoma are mainly associated with HRQoL, bother by disease, and needs for support. These results accentuate the need for attention for HRQoL in this population and for a multidisciplinary approach to pituitary care, as well as interventions to improve patients’ self-perceived health status in the chronic phase of prolactinoma treatment.

## Electronic supplementary material

Below is the link to the electronic supplementary material.

Supplementary file1 (PDF 435 kb)

## Data Availability

Data requests can be directed to DJL: D.J.Lobatto@lumc.nl.
