# Supplementary material for: Healthcare utilization and costs among prolactinoma patients: a cross-sectional study and analysis of determinants
Source: Pituitary. 2020 Oct 6;24(1):79–95. doi: 10.1007/s11102-020-01089-1 (PMC7864816; doi:10.1007/s11102-020-01089-1)
Supplement: Supplementary file 1 — Supplementary file1 (PDF 435 kb) [file 11102_2020_1089_MOESM1_ESM.pdf]

Healthcare utilization and costs among prolactinoma patients: a cross-sectional study and analysis of determinants

Supplements

Supplement 1. Characteristics of 111 patients with prolactinoma categorized by tumor size (missing data: n = 5)

	Microprolactinoma (N = 53)		Macroprolactinoma (N = 58)		P-value
**Demographic characteristics**					
Sex, N (%)					
Female	45	(84.9)	33	(56.9)	**0.001**
Age in years, mean (SD)	50.3	(14.7)	52.6	(13.0)	0.386
Female patients aged ≥50 years, N (% of female patients)	19	(42.2)	17	(51.5)	0.416
Marital status, N (%)					
Relationship/married	39	(73.6)	45	(78.9)	0.508
Education, N (%)					
Low	11	(20.8)	16	(28.1)	
Intermediate	16	(30.2)	11	(19.3)	
High	26	(49.1)	30	(52.6)	0.369
**Disease characteristics**					
Time since diagnosis in years, median (IQR)	16.6	(8.9-26.5)	13.2	(6.3-25.7)	0.462
Treatment, N (%)					
No treatment	3	(5.7)	0	-	
Medication only	44	(83.0)	37	(63.8)	
Surgery only	0	-	0	-	
Radiotherapy only	0	-	0	-	
Medication + surgery	6	(11.3)	14	(24.1)	
Medication + radiotherapy	0	-	1	(1.7)	
Surgery + radiotherapy	0	-	3	(5.2)	
Medication + surgery + radiotherapy	0	-	3	(5.2)	**0.018**
Endocrine status, N (%)					
Elevated prolactin level	23	(53.5)	12	(23.5)	**0.003**
Hypopituitarism	14	(26.4)	32	(55.2)	**0.002**
Adrenal insufficiency	3	(5.7)	14	(24.1)	**0.007**
**Self-reported health status**					
EQ-5D score, mean (SD)*	0.908	(0.099)	0.916	(0.068)	0.606
EQ-5D VAS, mean (SD)*	73.4	(21.6)	78.0	(17.5)	0.219
SF-36 PCS, mean (SD)*	48.5	(10.4)	49.1	(9.5)	0.736
SF-36 MCS, mean (SD)*	47.6	(12.0)	48.6	(11.5)	0.673
LBNQ-Pituitary Bother by disease total index score, mean (SD)†	16.0	(20.1)	15.8	(18.7)	0.967
LBNQ-Pituitary Needs for support total index score, mean (SD)†	17.8	(22.3)	15.7	(18.7)	0.603

n (number), SD (standard deviation), IQR (interquartile range), VAS (visual analogue scale), EQ-5D (EuroQoL), SF-36 (Short Form-36), LBNQ-Pituitary (Leiden Bother and Needs Questionnaire-Pituitary), MCS (mental component scale), PCS (physical component scale), ***bold*** *(P<0.05)*

** Higher scores indicate better HRQoL*

† Lower scores indicate lower disease burden

Due to rounding, not all percentages of the categorical variables add up to 100%

Supplement 2. Average healthcare utilization over the past 12 months in 111 patients with prolactinoma categorized by tumor size (missing: n = 5)

**Healthcare service**	**Microprolactinoma (N = 53)**		**Macroprolactinoma (N = 58)**		**P-value**
	Number of patients, %	Visits among those visiting, mean (SD)	Number of patients, %	Visits among those visiting, mean (SD)	
General practitioner	21 (39.6)	3.9 (4.3)	21 (36.2)	5.0 (5.7)	0.488
**Pituitary adenoma related medical specialists**					
Endocrinologist	43 (81.1)	2.1 (1.8)	53 (91.4)	1.9 (1.3)	0.935
Neurosurgeon	2 (3.8)	1.5 (0.7)	4 (6.9)	1.3 (0.5)	0.631
Ophthalmologist	14 (26.4)	2.5 (3.7)	15 (25.9)	1.9 (0.7)	0.605
ENT-specialist	1 (1.9)	1.0	3 (5.2)	2.0 (1.0)	0.223
Neurologist	5 (9.4)	1.4 (0.9)	2 (3.4)	1.5 (0.7)	0.286
Radiation oncologist	7 (13.2)	1.6 (0.8)	10 (17.2)	1.1 (0.3)	0.857
Cardiologist	5 (9.4)	2.6 (2.5)	8 (13.8)	1.4 (0.5)	0.717
Gynecologist	1 (1.9)	1.0†	0	-	†
Internist	6 (11.3)	3.5 (2.1)	2 (3.4)	3.5 (3.5)	0.175
Others	4 (7.5)	2.5 (1.3)	4 (6.9)	3.8 (1.9)	0.317
Total number of different specialists					
0	6 (11.3)	-	3 (5.2)	-	
1	26 (49.1)	1.7 (1.4)	27 (46.6)	1.5 (0.9)	
2	11 (20.8)	4.6 (4.6)	14 (24.1)	3.5 (1.7)	
3	5 (9.4)	6.2 (2.7)	7 (12.1)	6.4 (3.5)	
4 or more	5 (9.4)	13.4 (4.7)	7 (12.1)	7.9 (2.4)	0.768
**Occupational care**					
Occupational physician	6 (11.3)	6.3 (3.8)	3 (5.2)	4.0 (2.8)	0.089
**Mental healthcare**					
Psychologist/psychiatrist	9 (17.0)	11.8 (7.9)	6 (10.3)	8.0 (6.0)	0.159
**Allied health professionals**					
Physiotherapist	12 (22.6)	18.2 (23.7)	10 (17.2)	9.6 (7.4)	0.190
Dietician	5 (9.4)	3.2 (3.8)	3 (5.2)	5.3 (2.3)	0.919
Total number of different allied health professionals					
0	38 (71.7)	-	46 (79.3)	-	
1	13 (24.5)	9.38 (7.6)	11 (19.0)	7.6 (5.2)	
2	2 (3.8)	56.0 (50.9)	1 (1.7)	28.0†	0.595
**Emergency care**					
Ambulance rides	4 (7.5)	1.0 (0.0)	1 (1.7)	1.0†	†
Emergency department visit(s)	8 (15.1)	1.3 (0.5)	4 (6.9)	1.3 (0.5)	1.000
Hospital admission(s)	4 (7.5)	7.3 (1.0)*	2 (3.4)	12.0 (11.3)*	0.413
**Home care**					
Community nurse	1 (1.9)	13.0†^	0	-	†
Informal care	0	-	0	-	-
Household help	2 (3.8)	156.0 (73.5)^	0	-	†

n (number), SD (standard deviation), ENT (ear, nose and throat)
P-value based on number and frequency of visits
† No SD or P-value could be calculated because the number of patients in the categories was too low
** Hospital admissions are presented in days*
^ Community nurse, informal care, and household help are presented in hours

Supplement 3. Direct and indirect costs in euros (€) over the past 12 months in 111 patients with a prolactinoma, categorized by tumor size (missing: n = 5)

	Microprolactinoma (N = 53)		Macroprolactinoma (N = 58)		
**Medical costs**	**Number of patients (%)**	**Costs among those visiting, mean (SD)**	**Number of patients (%)**	**Costs among those visiting, mean (SD)**	**P-value**
**Medical costs of chronic specialist care**					
General practitioner	21 (39.6)	129 (140)	20 (34.5)	165 (188)	0.488
Specialist care	47 (88.7)	374 (421)	55 (94.8)	313 (269)	0.379
Occupational care	6 (11.3)	209 (126)	3 (5.2)	132 (93)	0.467
Mental healthcare^	9 (17.0)	754 (507)	6 (10.3)	512 (386)	0.341
Allied health professionals*	15 (28.3)	515 (740)	12 (20.7)	308 (254)	0.365
Total costs of chronic specialist care	49 (92.5)	735 (936)	57 (98.3)	483 (480)	0.093
**Medical costs of acute care**					
Ambulance rides	4 (7.5)	515 (0)	1 (1.7)	515†	†
Emergency department visits	8 (15.1)	324 (230)	4 (6.9)	324 (130)	1.000
Hospitalization	4 (7.5)	5355 (2618)	2 (3.4)	10472 (12117)	0.657
Total costs of acute care	9 (17.0)	2897 (3030)	5 (8.6)	4551 (8560)	0.601
**Home care costs**					
Home care#	2 (3.8)	11518 (5552)	0	-	†
Medical costs of chronic specialist and acute care	50 (94.3)	1242 (2050)	57 (98.3)	882 (2673)	0.441
Total medical costs	50 (94.3)	1703 (3806)	57 (98.3)	882 (2673)	0.196
**Medication costs**	**Number of patients (%)**	**Costs among those using medication, mean (SD)**	**Number of patients (%)**	**Costs among those using medication, mean (SD)**	**P-value**
Cabergoline	17 (32.1)	700 (888)	32 (55.2)	435 (393)	0.255
Quinagolide	8 (15.1)	323 (110)	4 (6.9)	310 (219)	0.896
Bromocriptine	3 (5.7)	77 (65)	1 (1.7)	19†	0.521
Total costs of dopamine agonists	27 (50.9)	545 (733)	37 (63.8)	410 (378)	0.341
Androgel	3 (5.7)	428 (383)	11 (19.0)	370 (182)	0.823
Contraceptives	2 (3.8)	25 (5)	3 (5.2)	47 (38)	0.491
Thyrax	6 (11.3)	33 (8)	22 (37.9)	34 (11)	0.901
Hydrocortisone	3 (5.7)	294 (234)	14 (24.1)	390 (309)	0.623
Genotropin	0	-	11 (19.0)	333 (1521)	†
Desmopressin	1 (1.9)	20†	2 (3.4)	383 (499)	0.658
Total costs of hormone replacement therapy	14 (26.4)	174 (284)	30 (51.7)	1595 (1926)	**<0.001**
Total medication costs	32 (60.4)	536 (698)	46 (79.3)	1370 (1652)	**0.003**
Overall costs	50 (94.3)	2046 (3760)	58 (100.0)	1954 (3044)	0.889

n (number), SD (standard deviation), ***bold*** *(P<0.05)*
** Physiotherapists and dieticians*
^ Psychiatrists and psychologists
# Community nurse, informal care, and household help
† No SD or P-value could be calculated because the number of patients in the categories was too low

Supplement 4a. Bother by disease and needs for support, as measured by the Leiden Bother and Needs Questionnaire for patients with pituitary disease, among 111 patients with a prolactinoma categorized by tumor size and treatment (missing: n = 5)

	Total cohort (N = 116)	Micro, no treatment or medication only (N = 47)	Micro, surgery and/or radiotherapy (N = 6)	Macro, no treatment or medication only (N = 37)	Macro, surgery and/or radiotherapy (N = 21)
**Bother by disease**	Mean (SD)	Mean (SD)	Mean (SD)	Mean (SD)	Mean (SD)
Physical & cognitive complaints	20.0 (22.9)	18.8 (22.9)	35.7 (39.8)	17.4 (19.5)	23.8 (23.8)
Mood	19.2 (24.1)	16.7 (22.9)	34.0 (36.8)	18.6 (23.5)	22.0 (24.9)
Negative illness perceptions	13.0 (18.3)	10.1 (14.7)	14.2 (18.8)	15.3 (21.0)	14.3 (20.7)
Sexual functioning	16.7 (22.8)	16.5 (22.1)	11.1 (18.0)	15.5 (24.7)	21.4 (20.7)
Social functioning	11.2 (21.3)	8.4 (19.3)	28.3 (34.7)	9.1 (21.6)	16.4 (19.8)
Total index score	15.8 (19.1)	14.5 (18.5)	26.9 (29.2)	13.4 (17.3)	19.9 (20.5)
**Needs for support**					
Physical & cognitive complaints	19.9 (24.6)	19.5 (26.1)	37.5 (41.7)	14.9 (18.9)	24.3 (22.7)
Mood	20.6 (26.5)	17.5 (25.1)	35.4 (39.9)	19.1 (25.8)	25.8 (26.9)
Negative illness perceptions	17.3 (22.3)	15.2 (19.5)	17.5 (22.3)	18.6 (25.3)	18.1 (22.8)
Sexual functioning	17.8 (26.0)	16.8 (24.6)	18.1 (20.0)	18.2 (29.3)	19.8 (27.6)
Social functioning	11.4 (21.6)	10.3 (20.6)	30.8 (39.0)	8.1 (21.0)	13.8 (16.8)
Total index score	16.7 (20.3)	16.2 (20.4)	29.6 (33.3)	13.3 (17.9)	20.1 (19.9)

n (number), SD (standard deviation), ***bold*** *(P <0.05)*
Higher scores indicate greater bother by disease and greater needs for support.

Supplement 4b. Bother by disease and needs for support, as measured by the Leiden Bother and Needs Questionnaire for patients with pituitary disease, among 111 patients with a prolactinoma categorized by tumor size (missing: N = 5)

	Total (N = 116)	Microprolactinoma (N = 53)	Macroprolactinoma (N = 58)	P-value*
**Bother by disease**	Mean (SD)	Mean (SD)	Mean (SD)	
Physical & cognitive complaints	20.0 (22.9)	20.7 (25.4)	19.7 (21.2)	0.863
Mood	19.2 (24.1)	18.7 (25.0)	19.8 (23.8)	0.997
Negative illness perceptions	13.0 (18.3)	10.6 (15.1)	14.9 (20.7)	0.335
Sexual functioning	16.7 (22.8)	15.9 (21.6)	17.7 (24.5)	0.828
Social functioning	11.2 (21.3)	10.7 (22.1)	11.7 (21.1)	0.977
Total index score	15.8 (19.1)	16.0 (20.1)	15.8 (18.7)	0.904
**Needs for support**				
Physical & cognitive complaints	19.9 (24.6)	21.6 (28.3)	18.2 (20.6)	0.573
Mood	20.6 (26.5)	19.6 (27.3)	21.6 (26.2)	0.938
Negative illness perceptions	17.3 (22.3)	15.5 (19.7)	18.4 (24.2)	0.639
Sexual functioning	17.8 (26.0)	17.0 (24.0)	18.8 (28.5)	0.600
Social functioning	11.4 (21.6)	12.6 (23.7)	10.1 (19.7)	0.296
Total index score	16.7 (20.3)	17.8 (22.3)	15.7 (18.7)	0.597

N (number), SD (standard deviation), ***bold*** *(P <0.05)*

** Corrected for age, sex, and education*

Higher scores indicate greater bother by disease and greater needs for support.
